# Better performance of deep learning pulmonary nodule detection using chest radiography with pixel level labels in reference to computed tomography: data quality matters

**DOI:** 10.1038/s41598-024-66530-y

**Published:** 2024-07-10

**Authors:** Jae Yong Kim, Wi-Sun Ryu, Dongmin Kim, Eun Young Kim

**Affiliations:** 1 Artificial Intelligence Research Center, JLK Inc., 5 Teheran-ro 33-gil, Seoul, Republic of Korea; 2 Department of Radiology, Incheon Sejong Hospital, 20, Gyeyangmunhwa-ro, Gyeyang-gu, Incheon, 21080 Republic of Korea

**Keywords:** Computer science, Radiography

## Abstract

Labeling errors can significantly impact the performance of deep learning models used for screening chest radiographs. The deep learning model for detecting pulmonary nodules is particularly vulnerable to such errors, mainly because normal chest radiographs and those with nodules obscured by ribs appear similar. Thus, high-quality datasets referred to chest computed tomography (CT) are required to prevent the misclassification of nodular chest radiographs as normal. From this perspective, a deep learning strategy employing chest radiography data with pixel-level annotations referencing chest CT scans may improve nodule detection and localization compared to image-level labels. We trained models using a National Institute of Health chest radiograph-based labeling dataset and an AI-HUB CT-based labeling dataset, employing DenseNet architecture with squeeze-and-excitation blocks. We developed four models to assess whether CT versus chest radiography and pixel-level versus image-level labeling would improve the deep learning model’s performance to detect nodules. The models' performance was evaluated using two external validation datasets. The AI-HUB dataset with image-level labeling outperformed the NIH dataset (AUC 0.88 vs 0.71 and 0.78 vs. 0.73 in two external datasets, respectively; both p < 0.001). However, the AI-HUB data annotated at the pixel level produced the best model (AUC 0.91 and 0.86 in external datasets), and in terms of nodule localization, it significantly outperformed models trained with image-level annotation data, with a Dice coefficient ranging from 0.36 to 0.58. Our findings underscore the importance of accurately labeled data in developing reliable deep learning algorithms for nodule detection in chest radiography.

## Introduction

Pulmonary disorders are leading global causes of morbidity, mortality, and health service utilization^1^. Chest radiography is the most commonly performed diagnostic test in everyday medial practice because of its accessibility, relative affordability, and widespread availability in outpatient clinics^2^. It is crucial for screening lung cancer, which is the leading cause of cancer-related deaths in both men and women in the United States^3^. The missed detection of pulmonary nodules or masses during chest radiography could lead to delayed diagnosis and management of both benign and malignant conditions, underscoring the importance of prompt reporting by radiologists for each image. However, prompt reporting is not always feasible due to the high volume of work in large healthcare facilities or the inexperience of radiologists in less-developed regions^3–5^.

Deep learning approaches has shown a remarkable success in recent years^6^. The integration of deep learning with convolutional neural networks in radiography has considerably improved the diagnosis of various pulmonary disorders, such as pulmonary tuberculosis, pneumonia, and pulmonary nodule^7–10^. Previous research utilized large datasets including National Institute of Health (NIH) Chest X-ray 14^11^, CheXpert^12^, and MIMIC-CXR^13^ for pulmonary nodule detection. However, the performance of these algorithms are highly constrained by the class imbalance of the dataset and label noise resulting from disease label extraction process^14,15^. Incorrect labels impede the generalization of predictive models and compromise the validity of model evaluation during training and testing^16^. Therefore, label cleaning is crucial to improve both model training and evaluation. Specifically for pulmonary nodule detection, these algorithms provide limited information on the exact location, which is important given the small and localized nature of pulmonary nodules^17^. Consequently, there is a demand for high-quality data referenced to chest computed tomography (CT) with pixel-level label for the enhanced performance of deep learning algorithms in pulmonary nodule detection^18^. Indeed, a recent study using 100 patients (47 with clinically significant pulmonary nodules/masses) with labelling referencing to chest CT demonstrated an excellent sensitivity of 85% for nodule detection^19^.

From the perspective of clinical implementation of a deep-learning algorithm for nodule detection on chest radiographs, a study using the National Lung Screening Trial dataset showed that the deep-learning algorithm achieved higher sensitivity (96.0%) and higher specificity (93.2%) compared to radiologists (sensitivity of 88.0% and specificity of 82.8%)^20^. Furthermore, in a study of 300 radiograph images with 216 pulmonary nodules confirmed by chest CT, the deep-learning algorithm improved the performance of early-career physicians in detecting nodules, although the algorithm’s performance was degraded in the lung apices, paramediastinal areas, and retrodiaphragmatic areas^21^.

In this study, we hypothesized that a deep learning algorithm utilizing chest radiography data with pixel-level labels referencing chest CT scans could improve the performance of nodule detection and localization compared to that using a dataset with only image-level labels. A deep learning algorithm for segmenting lung nodules on chest X-rays using pixel-level annotation data informed by chest CT scans may enhance the accuracy of lung nodule detection, which is crucial for early lung cancer detection. By learning from CT-annotated datasets, this method reduces false positives and improves diagnostic confidence, thereby streamlining the diagnostic process and potentially improving patient outcomes through early intervention.

## Assessing current literature

Several studies have explored the application of deep learning for detecting pulmonary nodules in chest radiographs. However, few studies have compared the performance of deep learning algorithms across datasets or employed pixel-level labeling for nodule localization. In this section, we review relevant studies that have utilized deep learning for the detection and segmentation of pulmonary nodules in chest radiographs.

### Pulmonary nodule classification

In an earlier phase, using a large public dataset comprising over 100,000 chest radiographs, Rajpurkar et al.^22^ developed a 121-layer convolutional neural network, named CheXNet, to classify 14 different pulmonary diseases. They reported that CheXNet outperformed the average performance of radiologists on a test dataset annotated by four practicing academic radiologists. Recently, Ait Nasser et al.^23^ proposed an ensemble learning method utilizing three different architectures (Xception, EfficientNet-B5, DenseNet-201) using 26,316 radiographs from VinDR-CXR and CheXpert datasets. Blais and Akhloufi^24^ trained multiple models, including DenseNet, ResNet, MobileNet and Xception using the binary relevance approach from the CheXpert dataset. They investigated performance differences according to the optimizer and found that the Xception model, trained with the ADAM optimizer, outperformed other models for all 14 diseases in the CheXpert dataset. Utilizing the NIH dataset, Rajpurkar et al.^14^ developed CheXNeXt, which classifies clinically important abnormalities in chest radiographs and provides a saliency map. In pulmonary nodule detection, the area under curve (AUC) of the model was 0.89, which is comparable to that of radiologists.

However, despite these studies achieving promising results in detecting pulmonary disease using deep learning, their robustness remains limited. This limitation arises because the labels of public datasets were derived from radiology reports using natural language processing algorithms, which may introduce noisy labels^25–27^. Furthermore, these studies did not provide precise information regarding the location of nodules. While saliency maps were once thought to approximate lesion locations, a recent study has shown that saliency maps correlate poorly with actual lesions^28^.

### Pulmonary nodule detection

To date, only a few studies have demonstrated the performance of deep learning algorithms in detecting and segmenting pulmonary nodule. Chiu et al.^29^ developed a deep learning model based on the You Only Look Once version 4 for detecting pulmonary nodules. They demonstrated that an AI model with a combination of preprocessing approaches achieved the highest sensitivity of 79%, with 3.04 false positives per image, indicating a low positive predictive value. Another study revealed that automatic detection using deep learning and CT-confirmed data achieved a sensitivity of 94.1% and a specificity of 83.3%^30^. These studies demonstrated the high efficiency of deep learning algorithms in detecting pulmonary nodules. However, given the small and ambiguous nature of pulmonary nodules, a segmentation algorithm that can precisely indicate the location and size of the nodules on chest radiographs would be extremely valuable, especially for physicians or radiologists with limited experience.

## Methods

This study was approved by the institutional review boards of JLK Inc, and Gachon University Gil Medical Center, and the exemption was obtained from institutional review boards for the using AI-HUB dataset. The informed consent was waived for this retrospective study using anonymized imaging data by institutional review boards of Gachon University Gil Medical Center. In this proof-of-concept study, we used all the data from a publicly available dataset for model training.

### Datasets

We utilized two distinct datasets to compare the performance of nodule classification and localization between data with noisy labels and data with clean labels based on chest CT scans. The datasets were randomly divided into training (70%), tuning (10%), and validation (20%) sets. We employed the NIH Chest X-ray 14 dataset^11^ as a source of large-scale data with noisy labels. This dataset comprises 112,120 frontal chest radiographs along with their accompanying text reports, retrospectively retrieved from the PACS database of the NIH Clinical Center. For abnormal cases, we extracted 5,156 chest radiographs labeled exclusively with nodules or masses. An equal number of normal cases were randomly selected to balance the number of abnormal cases. As the dataset with clean labels, we used chest X-ray data from AI-HUB, a public dataset from the Republic of Korea, collected retrospectively from a university hospital. The presence of nodules in these chest radiographs was confirmed by experienced thoracic radiologists using chest CT as the reference standard. Additionally, the nodule locations were annotated with a bounding box referencing the chest CT scans. We generated pixel-level mask images for model training by drawing ellipses within the bounding boxes. Out of the 3,500 chest radiographs of nodules, we used 3,177 as abnormal cases, excluding low-quality images. Similarly, 3,177 chest radiographs were randomly selected from a pool of 10,000 normal radiographs. Duplicate images from the same patients were excluded from the AI-HUB dataset.

Two distinct chest radiography datasets were used for external validation. Chest X-ray data were collected retrospectively from Gachon University Gil Medical Center (GMC), comprising 246 nodules and 440 normal cases. An experienced thoracic radiologist labeled the nodules by referencing chest CT scans and annotated the locations using pixel-level mask images. The other dataset was the publicly available VinBig dataset (VBD)^31^, consisting of 15,000 chest radiographs collected in Vietnam. Each chest radiographic lesion was annotated with a bounding box by three experienced radiologists. We selected 83 chest radiographs annotated by two or more radiologists as having a nodule or mass in the same location to serve as abnormal cases.

### Deep convolutional neural network structure and development

**Figure 1:**
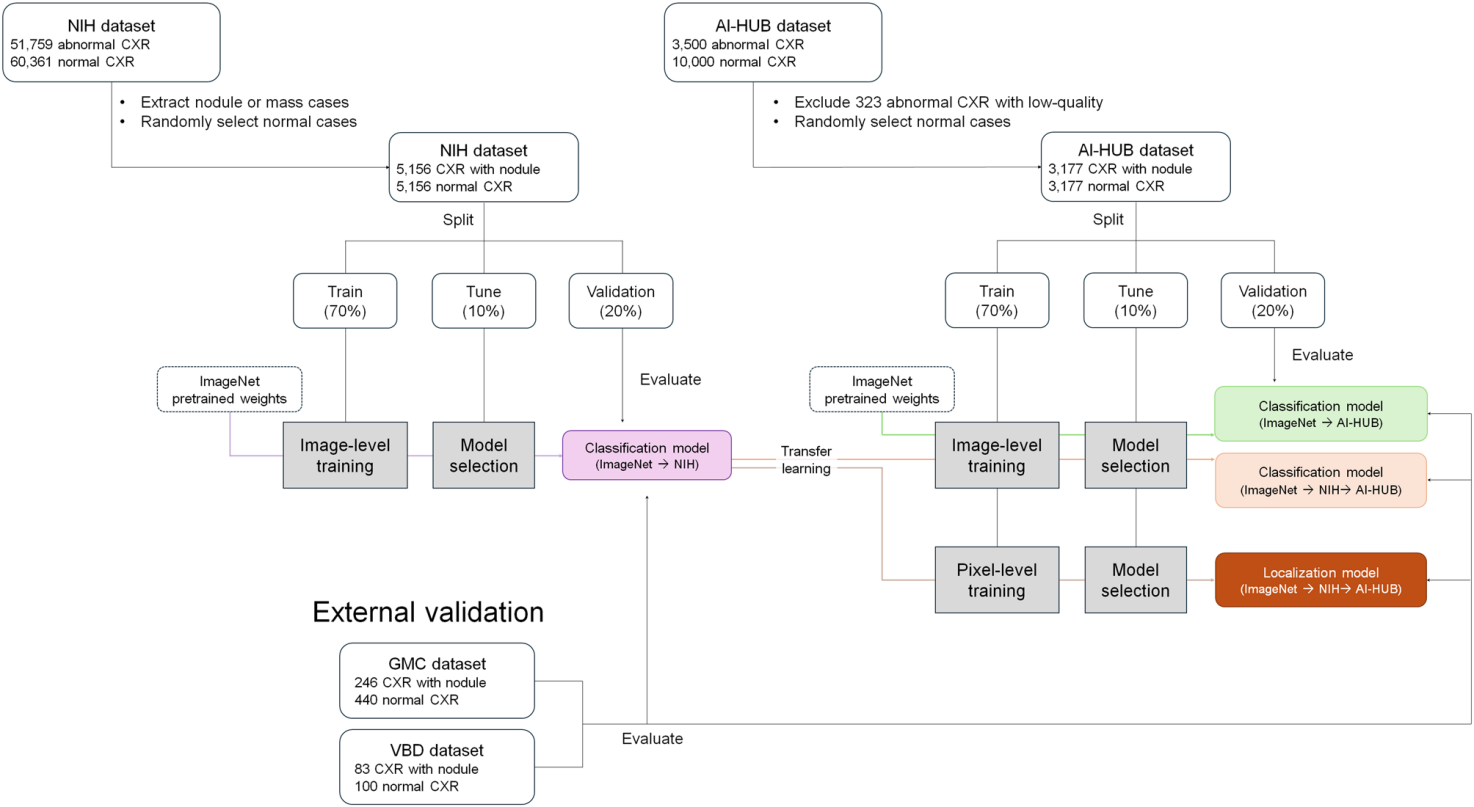
Study flow chart.

From the NIH and AI-HUB datasets, we randomly sampled normal and nodule or mass cases in a 1:1 ratio to address class imbalance (Table 1). We developed four models to explore whether (1) labeling based on chest CT versus chest radiography, and (2) pixel-level labeling versus image-level labeling, would improve deep learning performance in nodule detection. The first model, a deep learning network pre-trained on ImageNet, was transferred to the NIH dataset (Fig. 1). The second model, also pre-trained on ImageNet, was adapted to the AI-HUB dataset with image-level labeling. The third model, pre-trained on NIH data, was adapted to AI-HUB data with image-level labeling. The fourth model, pre-trained on NIH data, was adapted to AI-HUB data with pixel-level labeling.

**Table 1:** Training, tuning and validation datasets.

	Train		Tune		Validation			
	NIH	AI-HUB	NIH	AI-HUB	Internal		External	
					NIH	AI-HUB	GMC	VBD
No. of chest radiographs	7218	4446	1030	636	2064	1272	686	183
No. of normal radiographs	3609	2223	515	318	1032	636	440	100
No. of nodule radiographs	3609	2223	515	318	1032	636	246	83

The NIH and AI-HUB datasets were divided 7:1:2 into training, tuning, and validation data. NIH = National Institute of Health; GMC = Gachon Gil medical center; VBD = VinBig Data.

We designed two neural network architectures, as illustrated in Supplementary Fig. S1, utilizing DenseNet with squeeze-and-excitation (SE) blocks and sub-networks. The sub-network employs two convolutional layers to reduce the number of channels while maintaining the width and height of the input feature maps. Network 1, aimed at the classification task with image-level labeled data, is a standard DenseNet with SE blocks. It analyzes the input chest radiograph to estimate the probability of a nodule's presence, which ranges from 0 to 1. The final global average pooling layer of Network 1 transforms the output from the sub-network, a 16 × 16 feature map, into a single float to calculate the loss between the predicted probability and the true label. Additionally, Network 1 generates a class-activation map. Network 2, on the other hand, modifies Network 1 for use with pixel-level labeling, producing a single-channel probability map from the sub-network's output. We incorporated an upsampling layer between DenseNet and the sub-network to input a two-fold upscaled feature map into the sub-network. This adjustment was necessary because a 16 × 16 feature map produced by DenseNet with SE blocks was insufficient for accurately pinpointing the nodule location. The probability map identifies the nodule's location, with its maximum value indicating the likelihood of a nodule's presence. To train Network 2, we combined classification and localization losses. For localization loss, we employed the pixel-level binary cross-entropy mean. The localization loss function was calculated using the probability map generated by the deep learning model and a 32 × 32 resized nodule mask from the ground truth.

In the training phase, we cropped a single chest radiograph around lung area to prevent background information of original image from interfering with network training^32^. Then, we resized the cropped image to 512 × 512 pixels and fed it as an input to the network. Also, for superior performance and robustness, we applied contrast limited adaptive histogram equalization (CLAHE) and augmentation techniques such as horizontal flipping, shifting, scaling, rotating and shearing. We trained the network by using ADAM optimizer with a batch size of 16. We set the initial learning rate of 0.0001 and then multiplied by 0.1 at the 25th, 37th epoch. The experiments were based on Tensorflow v1.14.0 with Keras v2.3.0 and performed on an Intel® Xeon® Gold 5120 CPU @ 2.20 GHz and 2 NVIDIA Tesla V100 GPUs.

### Evaluation metrics

During the assessment of classification performance, each chest radiograph was categorized into one of four categories by comparing the predictions of the deep learning models with the image-level ground truth labels: true positive (TP), true negative (TN), false positive (FP), and false negative (FN). We employed three evaluation metrics: specificity (Sp), sensitivity (Se), and the area under the receiver operating characteristic (ROC) curve (AUC). To compare the AUC values of different deep learning models, we utilized DeLong's test for ROC curves^33^.

For the assessment of localization performance, we used the Dice coefficient score (DCS) between the class activation map or the probability map generated by deep learning models and ground truth mask annotated by thoracic radiologists. The metric is defined as follows:

$$DSC=\frac{2 \times true\, positive}{\left(True\, positive+False\, posotive\right)+(True\, positive+False\, negative)}$$

## Results

### Comparison of model performance on nodule detection

**Figure 2:**
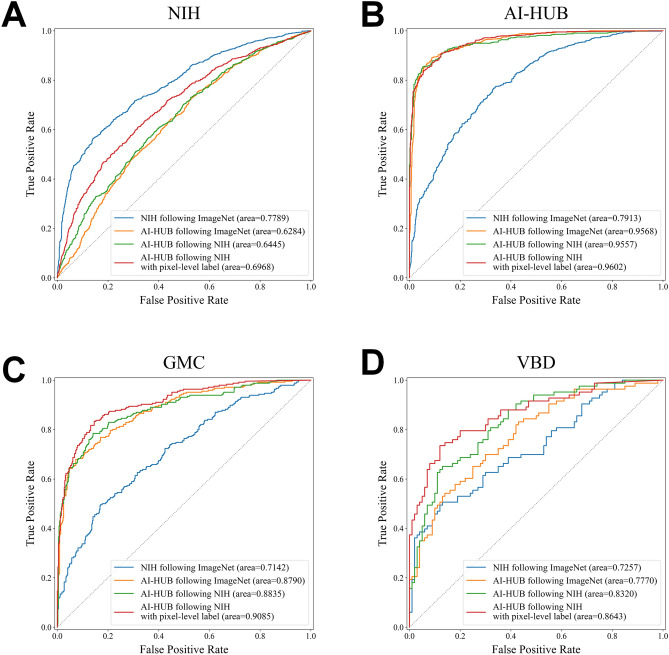
Receive operating characteristics curve of four models validated in internal and external datasets. (**A**) Validation using NIH data, (**B**) Validation using AI-HUB data, (**C**) Validation using GMC data, (**D**) Validation using VBD data. The NIH and VBD datasets were labeled using chest radiographs. Data labeling for AI-HUB and GMC was based on chest computed tomography. NIH = National Institute of Health; GMC = Gachon Gil Medical Center; VBD = VinBig data.

Table 2, Fig. 2, and Supplementary Figure S2 summarize the performance of deep learning models trained using different methods. Upon external validation, the model trained with the AI-HUB dataset (pre-trained on ImageNet) outperformed the model trained on the NIH dataset. In the GMC and VBD datasets, the AUCs for models trained with either AI-HUB or NIH were 0.88 and 0.78, versus 0.71 and 0.73, respectively (p < 0.001). Notably, the model trained with the AI-HUB dataset demonstrated superior performance on the dataset labeled with reference to chest CT compared to the dataset labeled with only chest radiographs (Fig. 1C and D). The model trained with the NIH dataset outperformed the model trained with the AI-HUB dataset using only their respective internal validation datasets (Fig. 1A). The performance of the model pre-trained with NIH dataset and then trained with the AI-HUB dataset showed improvement only in the VBD dataset (p < 0.001). Finally, the model trained with pixel-level labels from the AI-HUB dataset (pre-trained with the NIH dataset) exhibited the best performance across both internal and external validation datasets. For the GMC and VBD datasets, the AUCs for the model trained with pixel-level label were 0.91 and 0.86, respectively.

**Table 2:** Performances of four different deep learning algorithms in four different datasets.

Validation data	Image level data												Pixel level data			
	ImageNet → NIH				ImageNet → AI-HUB				ImageNet → NIH → AI-HUB				ImageNet → NIH → AI-HUB			
	AUC	Sp.	Se.	DSC	AUC	Sp.	Se.	DSC	AUC	Sp.	Se.	DSC	AUC	Sp.	Se.	DSC
NIH	0.78	0.82	0.60	NA	0.63	0.42	0.77	NA	0.64	0.56	0.63	NA	0.70	0.60	0.67	NA
AI-HUB	0.79	0.87	0.47	0.48	0.96	0.87	0.91	0.49	0.96	0.92	0.86	0.51	0.96	0.91	0.86	0.65
GMC	0.71	0.75	0.54	0.51	0.88	0.74	0.82	0.53	0.88	0.81	0.80	0.58	0.91	0.85	0.83	0.64
VBD	0.73	0.75	0.54	0.46	0.78	0.59	0.76	0.36	0.83	0.71	0.76	0.42	0.86	0.88	0.69	0.60

AUC = Area under receiver operating characteristic curve; Sp. = specificity; Se. = sensitivity; DSC = Dice similarity coefficient; NIH = National Institute of Health; GMC = Gachon Gil medical center; VBD = VinBig Data.

**Figure 3:**
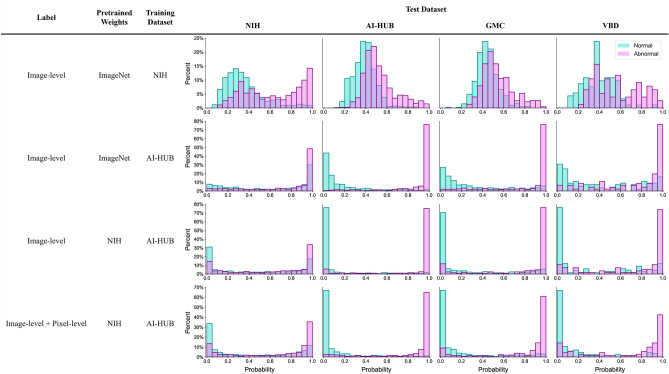
Probability distributions of models trained with different datasets for normal and nodule chest radiographs. NIH = National Institute of Health; GMC = Gachon Gil Medical Center; VBD = VinBig Data.

Figure 3 illustrates the probability distributions of the models for normal and abnormal (nodule) chest radiographs. The probability distributions for normal and abnormal cases generated by the model trained with NIH dataset showed overlap, especially in the external validation datasets, which indicates poor discriminative performance. In contrast, the model trained with the AI-HUB dataset effectively distinguished normal from abnormal cases. Moreover, the model trained with AI-HUB data exhibited an improved probability distribution, even when validated against the NIH dataset, despite having lower AUC values than the model trained with the NIH dataset.

In the GMC and VBD datasets, the deep learning model trained with pixel-level labels from the AI-HUB dataset (pre-trained on the NIH dataset) missed 42 and 26 nodules, respectively. The majority of the missed nodules (n = 51, 75%) were subcentimeter pulmonary nodules, and 42 (62%) were located in the apical and paramediastinal areas (Figure S3).

### Comparison of model performance on nodule localization

**Figure 4:**
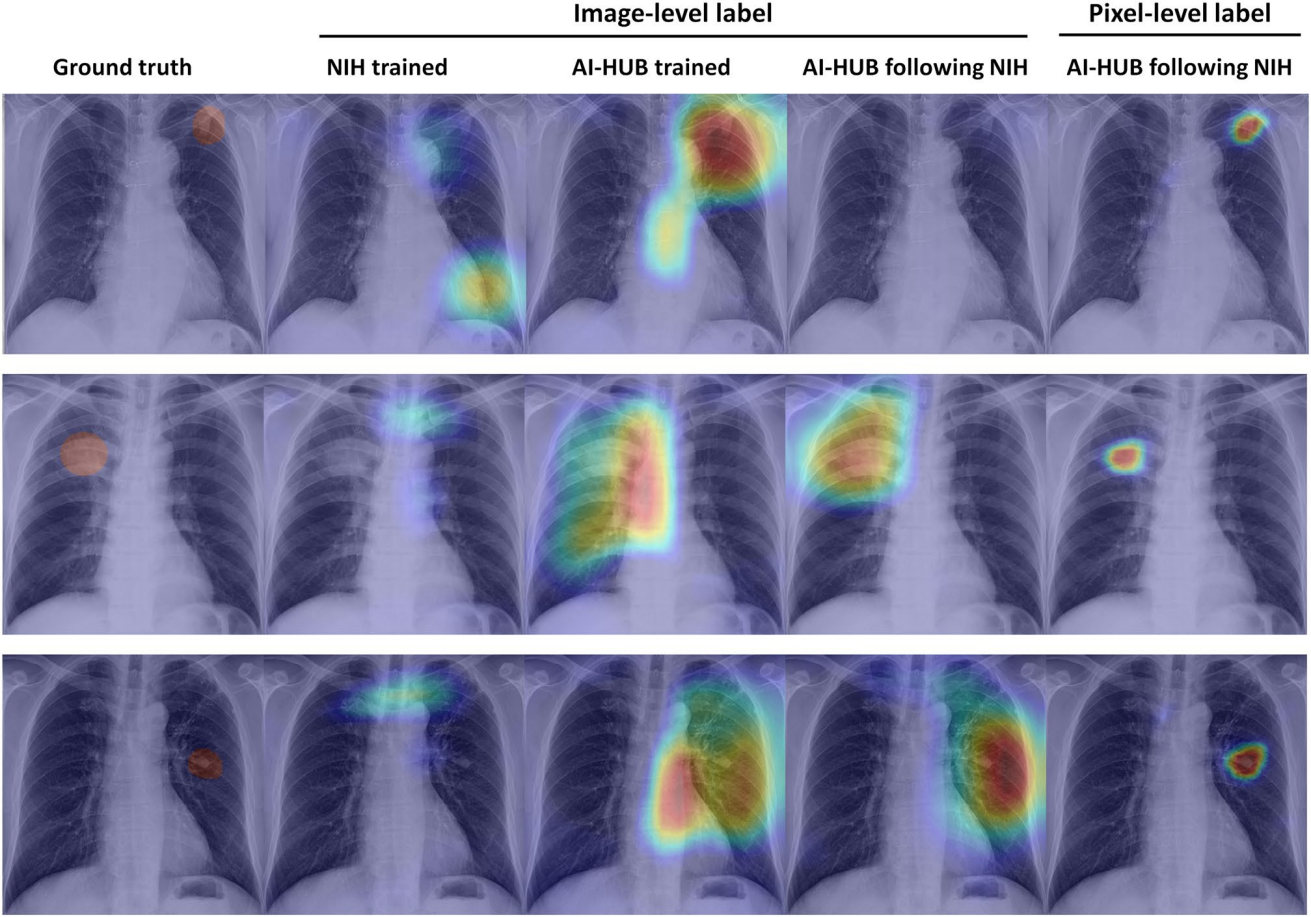
Representative radiographs, activation map, or heatmap derived from deep learning models. The first column indicates a thoracic radiologist's ground truth mask. The second, third, and fourth columns, respectively, represent activation maps obtained from models trained using NIH data, AI-HUB data, and AI-HUB data following NIH data with image-level label. The fifth column displays a heatmap obtained from a model trained with AI-HUB using NIH data labeled at the pixel level. NIH = National Institute of Health.

We compared the nodule localization performance of the models trained in testing on external validation datasets using pixel-level annotations (GMC dataset). The models trained with image-level labelling data (as shown in the first, second, and third columns of Table 2) exhibited DCS ranging from 0.36 to 0.58, which are comparable across models. However, the model trained with the AI-HUB dataset, annotated at the pixel level, demonstrated DCS greater than 0.60 across all validation datasets. Figure 4 displays representative radiographs that demonstrate the nodule localization performance of the trained models.

## Discussion

In this study, we found that a deep learning model trained on chest radiograph data, with labels referencing chest CT scans, performed better than a model trained on chest radiograph data without precise labeling for nodule detection. Furthermore, the model's performance was significantly improved by incorporating pixel-level annotations. The deep learning model, when trained with pixel-level labels, achieved more accurate localization of nodules on chest radiographs. To the best of our knowledge, this is the first study to report the use of a deep learning algorithm for segmenting lung nodules on chest X-rays utilizing pixel-level annotation data with referencing chest CT. This suggests that, even in large datasets, precise labeling and detailed information are crucial for developing an accurate deep learning model for the interpretation of medical images.

The NIH chest X-ray dataset, preprocessed using natural language processing, is known to contain incorrect and uncertain labels^15,22^. Several studies on the impact of label noise on the classification capabilities of convolutional neural networks (CNNs) have shown that CNNs can be resistant to significant label noise^34^. However, one study contended that deep learning–based computer-assisted diagnosis models are sensitive to label noise, arguing that diagnoses assisted by computers with inaccurate labels lack credibility^35^. Furthermore, it was revealed that the performance of deep learning models is inversely correlated with the proportion of data containing noisy labels^36^. Our findings, demonstrating that the deep learning model trained with more precise label data surpassed the performance of models trained with less precise data, align with those from previous research. Considering the critical implications of misclassifying medical images, precise data labeling is indispensable for developing deep learning models in medical imaging.

Although deep learning techniques have made significant advancements in the field of medical imaging, the 'domain shift problem' remains a considerable challenge. This problem refers to the phenomenon where a model's performance deteriorates when applied to data from a different domain^37^. It occurs because a deep learning model struggles to encapsulate the unique features specific to each vendor. In this study, the classification model trained with image-level labels was found to be vulnerable to the domain-shift problem, resulting in highly overlapping probability distributions for normal and abnormal cases. In contrast, the model trained with labels based on data referencing with chest CT exhibited a distinct difference in the distributions between nodular and normal chest radiographs, indicating the model's robustness, as corroborated by two external validation datasets.

The class activation map generated by a deep learning classification model offers a straightforward method for identifying the discriminative image regions that a neural network uses to recognize a specific class within an image. However, physicians often misinterpret these maps as directly indicating the lesion area^38^. Previous studies have demonstrated that class activation maps usually highlight disease-related regions, not necessarily the lesions themselves^39^. For example, the class activation map for pneumothorax detection generated by a deep learning model tends to focus around the chest tube, rather than on the pneumothorax itself^40^. A study utilizing the chest radiograph dataset for coronavirus disease 2019 (COVID-19) conclusively showed that the classification algorithm in deep learning often relies on 'shortcuts', such as a laterality marker, to differentiate between normal and abnormal cases^41^. In our study, the deep learning model was trained using two loss functions: nodule classification and localization, making it potentially less prone to the 'shortcuts' problem. This is supported by a higher DSC in the model trained with pixel-level data. Given the often small size of nodules on chest radiographs, providing precise localization alongside accurate classification could be highly beneficial in daily clinical practice.

A few studies have demonstrated the efficacy of commercial software solutions in detecting pulmonary nodules. In a sample of 100 patients, a computer-aided diagnosis software utilizing a deep learning approach achieved an AUC of 0.91^19^. Additionally, another study validated a different solution in 5,485 participants, where the sensitivity and specificity of the AI algorithm for detecting pulmonary nodules were 86.2% (95% CI 77.8–94.6%) and 85.0% (95% CI 81.9–88.1%), respectively^20^. In the present study, our deep learning algorithm achieved sensitivity and specificity comparable to those in two external validation datasets, underscoring the clinical utility of our algorithm.

The deep learning algorithm for detecting pulmonary nodules on chest radiographs offers significant technical advancements over traditional radiographic procedures. It provides enhanced sensitivity and specificity, reducing the likelihood of false negatives, and ensures more accurate diagnoses^42^. By delivering consistent and reproducible results, the algorithm minimizes diagnostic variability and provides reliable detection regardless of individual radiologist differences^43^. Additionally, the algorithm's ability to analyze images rapidly improves efficiency and reduces radiologist workload, enabling quicker clinical decision-making^44^. These benefits collectively enhance the accuracy and speed of pulmonary nodule detection, ultimately leading to improved patient outcomes.

Our findings should be interpreted with caution. In this study, the deep learning models were validated using two distinct external datasets. Due to the AI-HUB dataset and two external datasets collected from the Asian population, the observed performance difference in this study could be attributable, in part, to the ethnic variation of nodules. The transfer learning from NIH to AI-HUB may result in the overfitting of the algorithm. Although the model that utilized transfer learning exhibited better performance on the GMC and VBD datasets, the differing ethnic backgrounds between the NIH dataset and those of AI-HUB, GMC, and VBD prevent us from ruling out the possibility of overfitting. Thus, our results have limited generalizability to other ethnic backgrounds.

The detection of pulmonary nodules on chest radiographs is crucial because they may indicate pulmonary cancer. However, this is often difficult, and radiologists frequently miss pulmonary nodules^45^. Our findings indicate that accurately labeled data are required to train robust and reliable deep learning nodule detection models. Furthermore, the deep learning model trained with pixel-level data can provide precise information on nodule location.

### Supplementary Information

Supplementary Information.

## Data Availability

The NIH chest radiographs that support the findings of this study are publicly available at https://nihcc.app.box.com/v/ChestXray-NIHCC. And the VinBig dataset is publicly available at https://physionet.org/content/vindr-pcxr/1.0.0/.
