# Supplementary material for: Better performance of deep learning pulmonary nodule detection using chest radiography with pixel level labels in reference to computed tomography: data quality matters
Source: Sci Rep. 2024 Jul 10;14:15967. doi: 10.1038/s41598-024-66530-y (PMC11237128; doi:10.1038/s41598-024-66530-y)
Supplement: Supplementary file 1 — Supplementary Information. [file 41598_2024_66530_MOESM1_ESM.docx]

**Supplementary Information**

**Better performance of deep learning pulmonary nodule detection using chest radiography with reference to computed tomography: data quality is matter**

Jae Yong Kim, MSc.^1^ Wi-Sun Ryu, MD, PhD^1^*****, Dongmin Kim, PhD^1^,

Eun Young Kim, MD, PhD^2^*****

**Affiliations**

^1^Artificial Intelligence Research Center, JLK Inc., Seoul, Republic of Korea

^2^Department of Radiology, Gil Medical Center, Gachon University College of Medicine, Incheon, Republic of Korea

*** Corresponding author**

Wi-Sun Ryu, MD, PhD

Chief Medical Officer, JLK Inc.

Address: Artificial Intelligence Research Center, JLK Inc., 5 Teheran-ro 33-gil, Seoul, Republic of Korea

E-mail: [wisunryu@gmail.com](mailto:wisunryu@gmail.com)

Eun Young Kim, MD, PhD

Professor, Department of Radiology, Gil Medical Center, Gachon University College of Medicine

Address: Department of Radiology, Gil Medical Center, Gachon University College of Medicine, Namdong-daero 774 beon-gil, Namdong-gu, Incheon 21565, Republic of Korea

E-mail: [oneshot0229@gmail.com](mailto:oneshot0229@gmail.com)


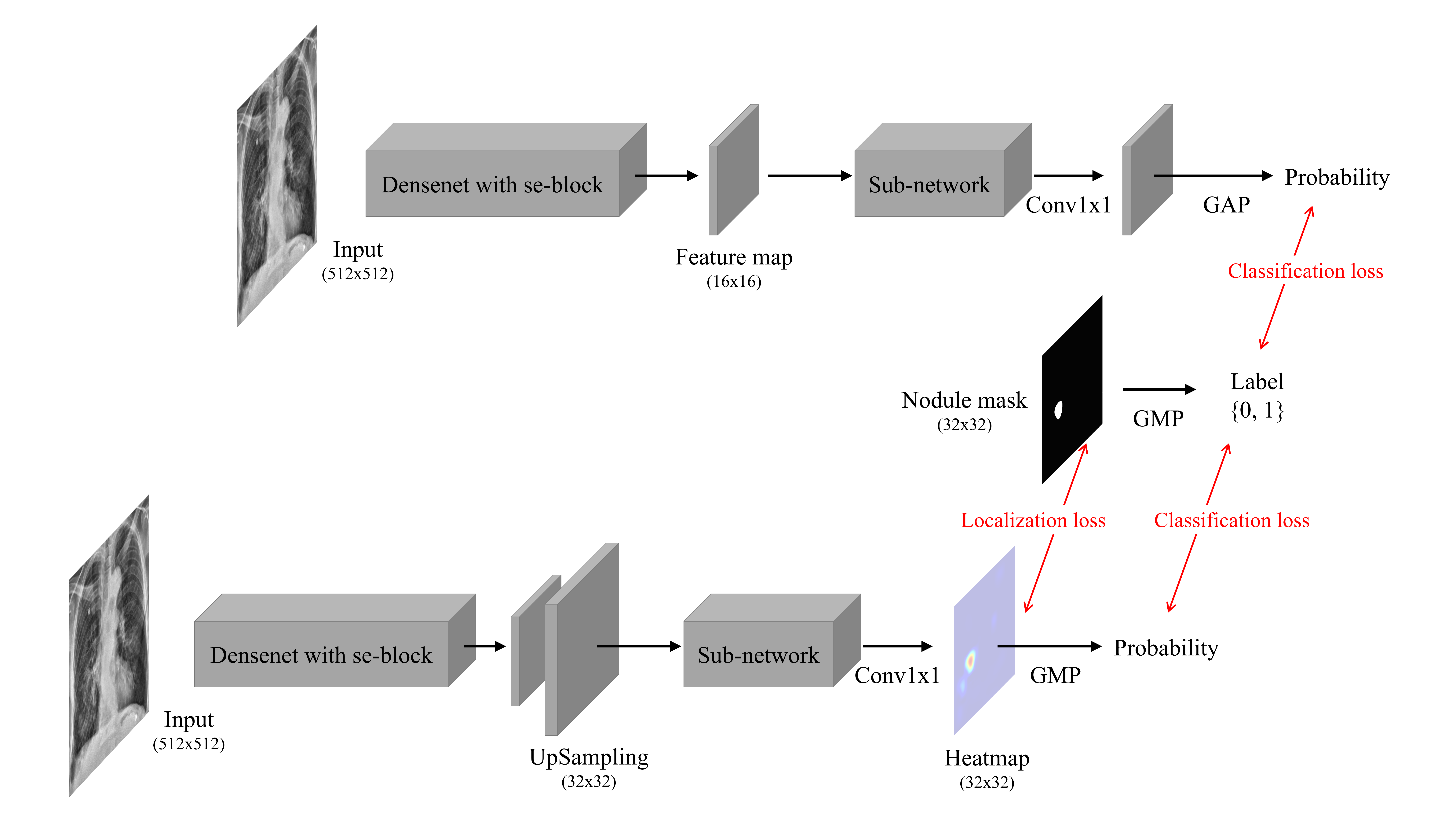


**Supplementary Figure S1. Deep learning network architecture for training.**

**
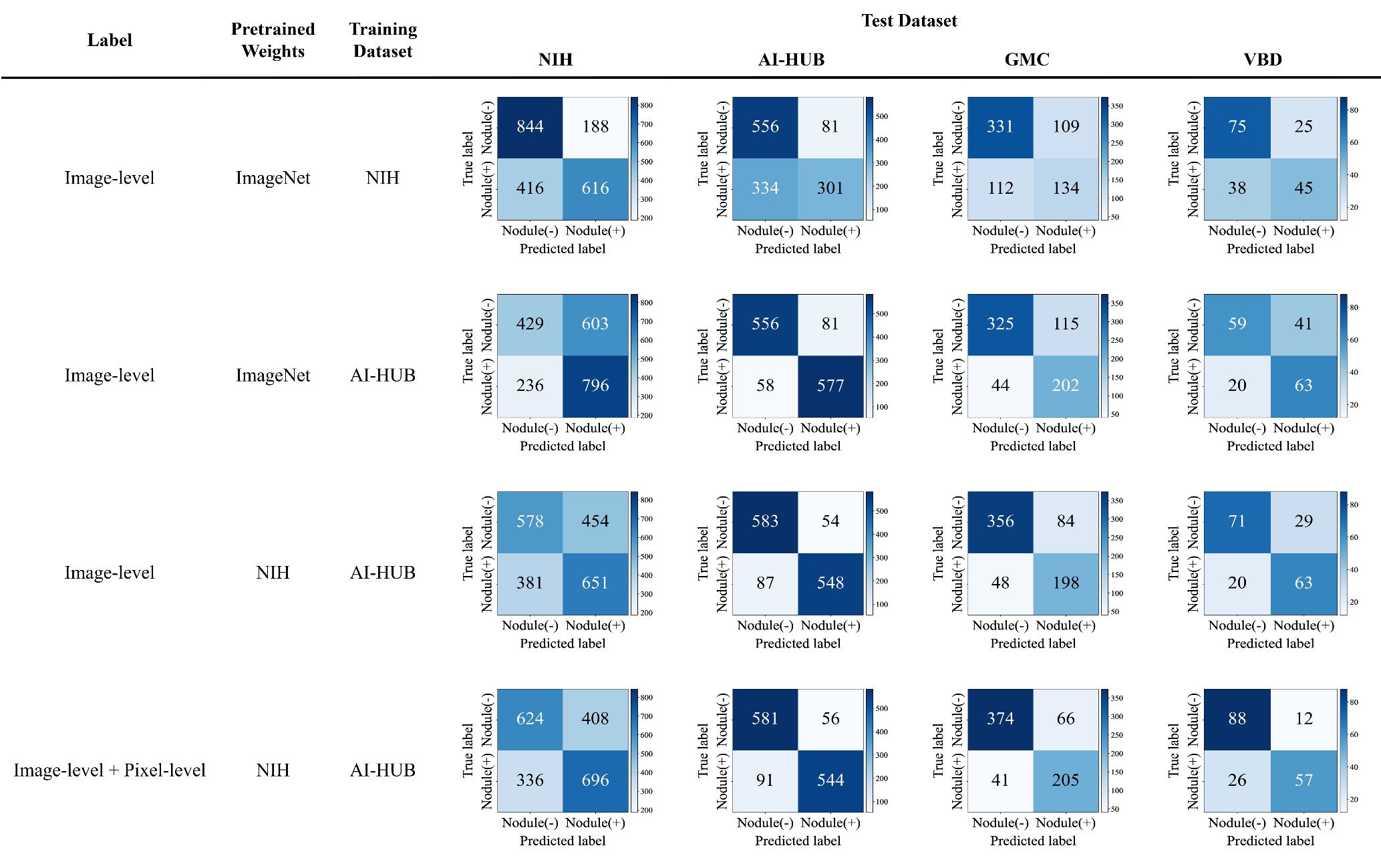
**

**Supplementary Figure S2. Confusion matrix for four different test datasets.** GMC=Gachon Gil medical center; VBD=VinBigData


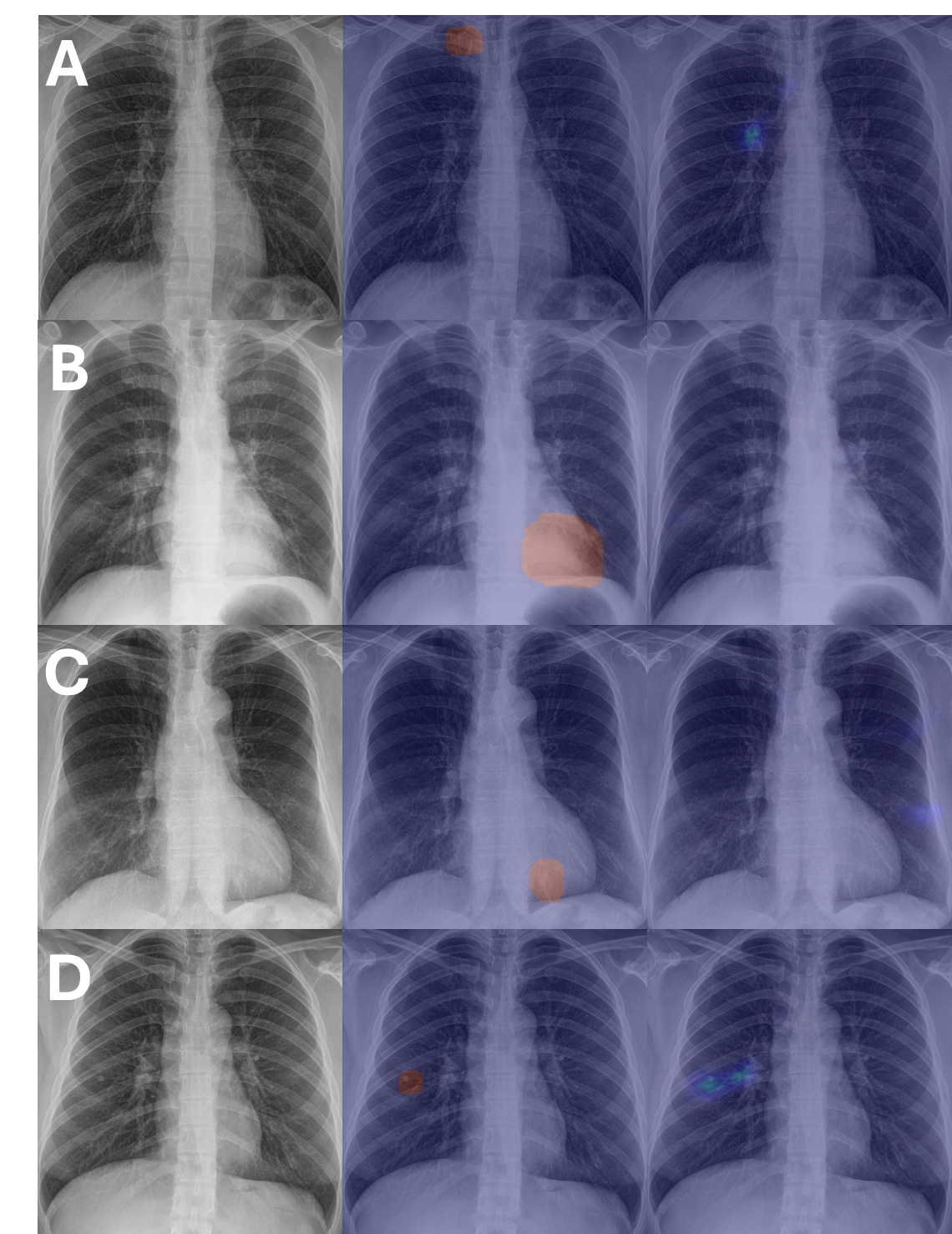


**Supplementary Figure S3. Representative cases of false negative.** Deep learning algorithm trained on the AI-HUB dataset (pretrained on NIH dataset). (A) A nodule in the apical region. (B and C) Nodules at paramediastinal area. (D) A subcentimeter nodule.
